# CLARITY for High-resolution Imaging and Quantification of Vasculature in the Whole Mouse Brain

**DOI:** 10.14336/AD.2017.0613

**Published:** 2018-04-01

**Authors:** Lin-Yuan Zhang, Pan Lin, Jiaji Pan, Yuanyuan Ma, Zhenyu Wei, Lu Jiang, Liping Wang, Yaying Song, Yongting Wang, Zhijun Zhang, Kunlin Jin, Qian Wang, Guo-Yuan Yang

**Affiliations:** ^1^Department of Neurology, Ruijin Hospital, Shanghai Jiao Tong University School of Medicine, Shanghai, China; ^2^Medical Image Computing Lab and; ^3^Neuroscience and Neuroengineering Research Center, Med-X Research Institute and School of Biomedical Engineering, Shanghai Jiao Tong University, Shanghai 200030, China; ^4^Shanghai Ninth People’s Hospital, Shanghai Jiao Tong University School of Medicine, Shanghai 201999, China; ^5^Department of Pharmacology and Neuroscience, University of North Texas Health Science Center, TX76107, USA

**Keywords:** brain, clarity, imaging process, mouse, vasculature

## Abstract

Elucidating the normal structure and distribution of cerebral vascular system is fundamental for understanding its function. However, studies on visualization and whole-brain quantification of vasculature with cellular resolution are limited. Here, we explored the structure of vasculature at the whole-brain level using the newly developed CLARITY technique. Adult male C57BL/6J mice undergoing transient middle cerebral artery occlusion and Tie2-RFP transgenic mice were used. Whole mouse brains were extracted for CLARITY processing. Immunostaining was performed to label vessels. Customized MATLAB code was used for image processing and quantification. Three-dimensional images were visualized using the Vaa3D software. Our results showed that whole mouse brain became transparent using the CLARITY method. Three-dimensional imaging and visualization of vasculature were achieved at the whole-brain level with a 1-μm voxel resolution. The quantitative results showed that the fractional vascular volume was 0.018 ± 0.004 mm^3^ per mm^3^, the normalized vascular length was 0.44 ± 0.04 m per mm^3^, and the mean diameter of the microvessels was 4.25 ± 0.08 μm. Furthermore, a decrease in the fractional vascular volume and a decrease in the normalized vascular length were found in the penumbra of ischemic mice compared to controls (*p* < 0.05). In conclusion, CLARITY provides a novel approach for mapping vasculature in the whole mouse brain at cellular resolution. CLARITY-optimized algorithms facilitate the assessment of structural change in vasculature after brain injury.

Cerebral blood vessels are channels for supplying oxygen, glucose and other nutrients to and removing wastes from the brain tissue. Numerous studies have demonstrated that the cerebral vasculature is an extremely important part for the brain survival and function [1-3]. Our previous study showed that alterations in vascular structure and distribution were associated with cerebral hemodynamic changes in rats following ischemic stroke [4]. Furthermore, recent studies unveiled an intimate structural and functional relationship between brain vasculature and other brain cells, with the formation of a functional domain termed the neurovascular unit [5]. Cerebral vascular density is closely related to neurological functional recovery after ischemic stroke [6]. Therefore, elucidating the spatial structure and distribution of the cerebral vasculature is a basis for understanding brain function. However, studies on visualization and quantification of the vasculature in the whole mouse brain at a cellular resolution are limited.

Conventional methods for evaluating tissue structure rely on histological sections and immunohistochemistry techniques. The brain sections are typically less than 50 μm in thickness to allow for antibody penetration. The thin sections also facilitate cellular and molecular observation by reducing light scattering. However, this could induce potential bias in quantification and interpretation due to insufficient spatial sampling [7]. To obtain unbiased information from whole-brain, new approaches have been proposed for whole brain imaging at high resolutions. One approach was a combination of block-face microscopy and tissue sectioning, and included two-photon tissue cytometry [8], serial two-photon (STP) tomography [9], knife-edge scanning microscopy (KESM) [10], and micro-optical sectioning tomography (MOST) [11]. The MOST technique could visualize and quantify cells and blood vessels in the whole mouse brain at 1-μm voxel resolution [12]. The MOST technique has been used to analyze the vascular distribution in the mouse barrel cortex [13]. However, the procedure was time-consuming, requiring approximately 19 days accomplishing simultaneously sectioning and imaging of one mouse brain [11-14].

Conversely, optical clearing approaches enable whole mouse brain imaging at a cellular resolution by using microscopy without mechanical sectioning. Tissue clearing was achieved by matching the refractive index of the tissue to the clearing reagent. The clearing reagents included Sca*l*eA2 [15], ClearT2 [16], 3DISCO [7, 17, 18], SeeDB [19, 20], CUBIC [21-23], and iDISCO [24]. 3DISCO was applied to visualize and quantify axonal regeneration and glial reaction after acute spinal cord injury in an unsectioned spinal cord from an adult mouse [7]. However, the drawback of these methods was the limited depth of exogenous antibody penetration into the brain. To overcome challenge, a new clearing technique termed CLARITY was developed. The technique transformed the entire tissue into a hydrogel-hybridized form to preserve the tissue architecture, protein and nuclear acid molecules, and endogenous fluorescence [25-27]. CLARITY not only made the brain tissues transparent but also allowed antibody diffusion deep into the brain parenchyma by removing lipid bilayers during the procedure. Structural and molecular information from the whole brain was obtained by imaging at a cellular resolution. The CLARITY technique has been used to image the whole brain and spinal cord to assess axonal pathology in a mouse experimental autoimmune encephalomyelitis model [28]. However, CLARITY technique has not been used to visualize the entire brain vasculature. Furthermore, computational processing compatible for CLARITY imaging of vasculature should be optimized to analyze the 3-dimensional vasculature structure of the mouse brain.

In the present study, we mapped the vascular network at the whole-brain level with cellular resolution using the CLARITY technique and extracted spatial information from cerebral vascular system. In addition, we validated CLARITY in the evaluations of vascular aberration in an ischemic mouse model.

## MATERIALS AND METHODS

### Experimental animals

The animal experimental procedure was approved by the Institutional Animal Care and Use Committee (IACUC) of Shanghai Jiao Tong University, Shanghai, China. We performed animal experiments in accordance with the guidelines of The Laboratory Animal Resource Center, Shanghai Jiao Tong University. Ten-week-old male wild type C57BL/6J mice (Sippr-BK, Shanghai, China) and B6.Cg-Tg (Tie2-RFP)1Ywa/J mice (Jackson Laboratory, Bar Harbor, ME, USA) weighting 25-30 grams were used in this study. Tie2-RFP transgenic mice express uniform red fluorescence in endothelial cells.

### Transient middle cerebral artery occlusion

Adult male wild-type C57BL/6J mice were used for the transient middle cerebral artery occlusion (tMCAO) surgery. The process was described previously [29]. Briefly, the mice were anesthetized with ketamine/ xylazine (100 mg/10 mg/kg, Sigma, St. Louis, MO). During the surgery, the body temperature was maintained at 37 ± 0.5 °C using a heating pad (RWD life science, Shenzhen, China). The left common carotid artery, external carotid artery, and internal carotid artery were isolated after midline inclusion. A nylon suture with a 6-0 silicone-coated tip (Covidien, Mansfield, MA, USA) was gently inserted from the external carotid artery to the internal carotid artery ending at the opening of the MCA to induce brain ischemia. Ninety minutes later, the suture was withdrawn to allow reperfusion. Surface cerebral blood flow (CBF) was monitored with a laser Doppler flowmetry (Moor Instruments, Axminster, Devon, UK). The suture was withdrawn immediately after insertion in the internal carotid artery in the sham mice. Mice were sacrificed 24 hours after reperfusion.

### CLARITY

All mouse brain clearing procedures were performed according to the optimized CLARITY protocol [30]. In brief, animals were deeply anesthetized with an intraperitoneal injection of ketamine/xylazine (100 mg/10 mg/kg, Sigma-Aldrich, St. Louis, MO) and perfused transcardially with ice-cold 0.1 M phosphate buffer saline (PBS) and then 4% paraformaldehyde. Subsequently, each mouse brain was rapidly extracted. The brain tissue was incubated in ice cold hydrogel solution (4% acrylamide and 0.25% VA-044 in 0.1 M PBS) at 4°C for 2 days followed by hydrogel polymerization at 37 °C for 4-5 hours. Residual oxygen was removed to ensure adequate hydrogel polymerization. The whole brain was then extracted from solidified hydrogel carefully and washed in 8% sodium dodecyl sulfate (SDS)/0.1 M PBS clearing solution with shaking at 60 °C to remove the lipid bilayer. The clearing solution was refreshed every day until the brain became completely transparent. Once the brain was clarified, 0.1 M PBS/0.1% TritonX-100 (PBST) was used for 3 days to remove residual SDS. PBST was refreshed every 6 hours. The tissue was then incubated in refractive index matching solution (RIMS, 88% HistodenZ, Sigma-Aldrich, St. Louis, MO, USA) for 12 hours at room temperature for refractive index homogenization. The whole brain was mounted to a home-built chamber filled with RIMS solution for imaging. For immunostaining, the whole brain was cut coronally into 1-mm-slices. After being cleared in 8% SDS solution with shaking at 60 °C and being washed in PBST, the brain slices were incubated with lectin (1:200, Vector laboratories, Youngstown, OH, USA) or mouse claudin-5 primary antibody (1:200, Invitrogen, Carlsbad, CA, USA) for 3 days at 37°C with shaking. For claudin-5 immunostaining, samples were further incubated in Alexa Fluor 555 donkey anti-mouse secondary antibody (1:200, Invitrogen, Carlsbad, CA) at 37 °C for an additional 2 days. Subsequently, the samples were washed in PBST at 37 °C for 24 hours followed by incubation in RIMS solution for 1 hour at room temperature before sample mounting. The samples were protected from light during all CLARITY steps.

### Imaging

Vasculature of whole mouse brain was imaged using Nikon A1RMP confocal laser scanning microscopy (Nikon Instruments Inc., Tokyo) equipped with a 40× water-immersion objective (Nikon CFI Apo NIR, numerical aperture = 0.8, working distance = 3.5 mm) at 543-nm wavelength excitation. The imaging depth was 3.27 mm with a voxel size of 0.62 μm×0.62 μm×1.38 μm. Furthermore, 1-mm-thick mouse brain slices stained with lectin-FITC and claudin-5 were imaged using a 16× water-immersion objective (Nikon CFI75 Achro LWD, numerical aperture = 0.8, working distance = 3.0 mm) and 488-nm and 543-nm wavelength excitation. The vascular selected chosen for quantification were obtained from the penumbra region in tMCAO and its counterpart in the controls. The imaging volume was 504 μm×504 μm×886 μm with a voxel size of 0.99 μm×0.99 μm×2.00 μm. The image resolution was 512×512.

### Image preprocessing and segmentation

**Figure 1.**
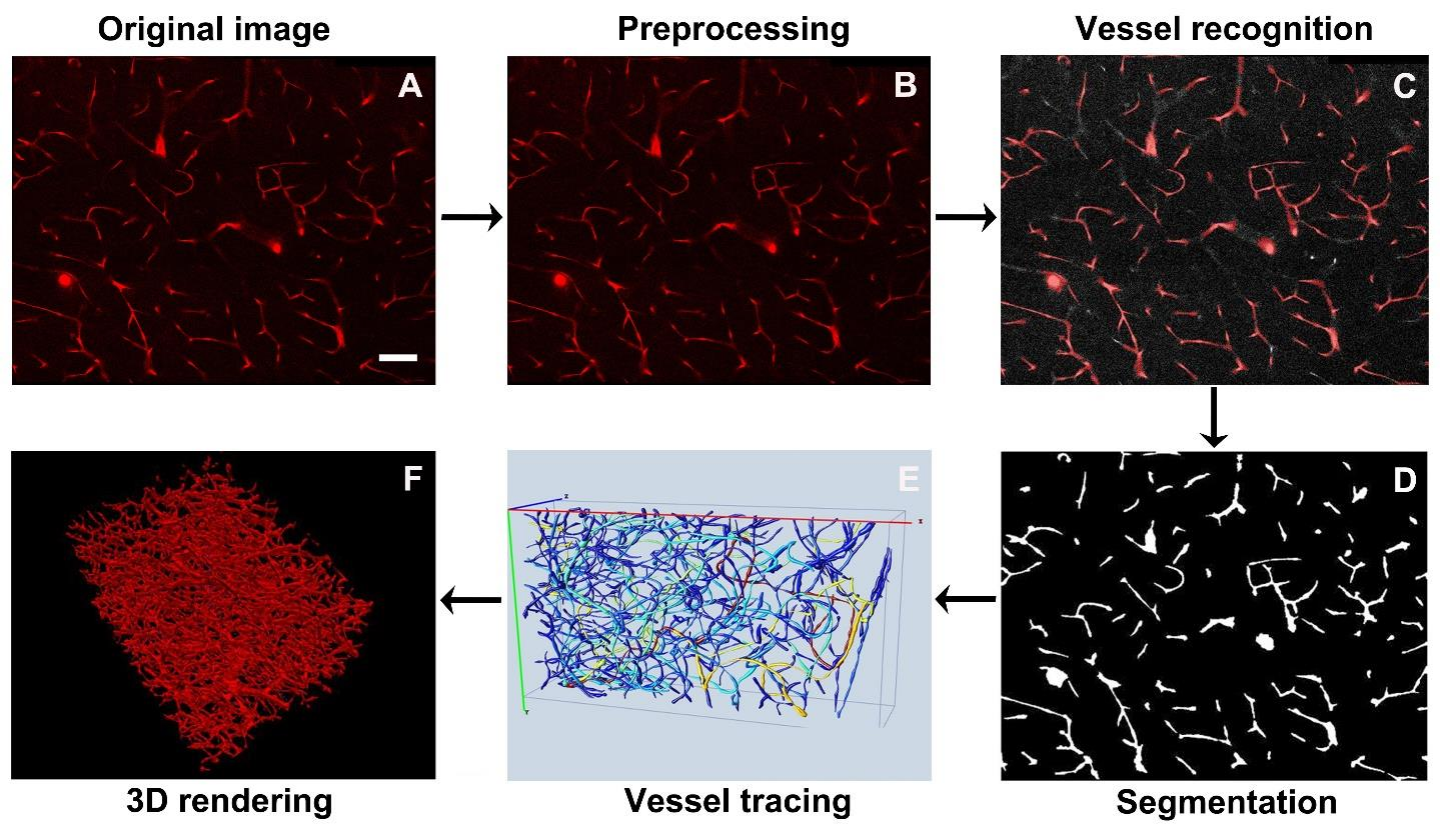
Processing procedures of clarified sample image. A) Original image obtained from the clarified sample. Scale bar=100 μm. B) Image preprocessing to uniform background intensity of the image. C) Vessel recognition by 3D Canny edge detection and morphology operation. D) Image segmentation and binarization. E) Vessel tracing using Vaa3D software. F) Visualization of 3D rendering images was performed by Vaa3D.

The processing flow of images is shown in Figure 1. The code is provided in the Supplementary materials. The original images were processed using MATLAB (Mathworks, Natick, MA, USA). Briefly, the images were cropped to exclude the useless margin in the first input step. Considering the decreased brightness of the original images with the imaging depth in the brain slices, correction of illumination along the imaging depth was performed. The pixel intensity of each image along the z-axis was revised using histogram matching method. The illumination intensity of each image Along the x- and y-axis was revised using L= (m*x)/p. In the formula, x and L indicated the intensity before and after correction, respectively; p denotes the mean value curve along the x- and y-axis, and m denotes the total mean value. The median filtering was applied to the images to correct the uneven background intensity value. Vessels were binarized using Otsu thresholding. Subsequently, 3D canny edge detection was used to extract the outline of the vessels and the morphology operation was performed to fill holes.

### Vessel Tracing

Tracing of the vasculature was performed using Vaa3D (www.vaa3d.org) [31]. In brief, the preprocessed image stacks were reshaped into a physical size. Tracing seeds were automatically localized at the center of the isolated regions using the pixel connectivity analysis. The blood vessels were identified based on the hierarchical pruning of a gray-weighted distance tree [32]. The results were exported into a SWC-formatted file. The diameter of a vessel was dynamically estimated using a radius-adjustable spherical region centered at the point on the centerline. The radius of the sphere was determined when 0.1% of the image voxels within this sphere were darker than the average voxel intensity of the entire image [33]. The tracing performance was evaluated by the recall and the precision value. The manually labeled results were used as a reference to examine the automatic tracing results. The recall represented the tracing ratio among all of the real blood vessels, and the precision represented the correct blood vessels among all of the traced vessels.

### Quantitative analysis

Quantitative analyses were performed using a customized MATLAB code. The histogram distribution of the vascular length corresponding to different diameters was automatically computed. The quantitative parameters of vessels were in accordance with pervious definitions [12, 13, 34]. The vascular density was defined as the fraction of vasculature volume that accounted for the total imaging volume. The normalized vascular length was also calculated as the total vascular length accounting for the total imaging volume. In addition, a vessel with a diameter less than 6 μm was classified as a microvessel according to previous studies [12, 34]. The average diameter of microvessels was also calculated.

### Statistical analysis

Descriptive statistics are presented as the means ± standard deviation. For comparison of the fractional vascular volume between different groups, student’s *t*-test was used. Statistical analyses were performed using the SPSS software (version 17.0, SPSS, Chicago, IL, USA). *p* < 0.05 was considered statistically significant.

## RESULTS

### Whole adult mouse brain transparency using CLARITY

**Figure 2.**
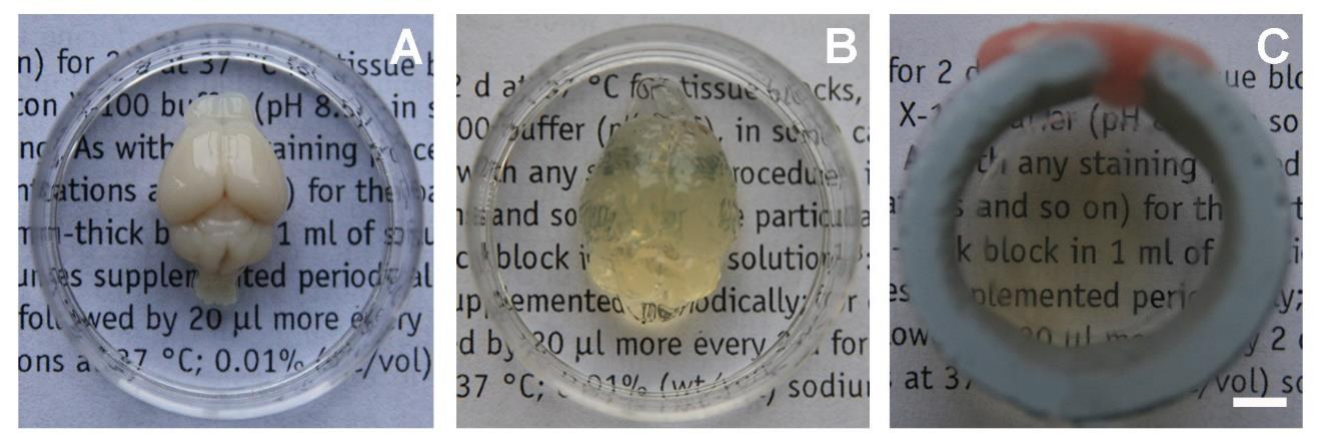
CLARITY renders whole adult mouse brain transparent. A) Whole adult mouse brain before CLARITY process. B) Whole adult mouse brain after removing lipid bilayers. C) Whole adult mouse brain after refractive index matching. Scale bar=1 mm.

Consistent with a previous report [27], we achieved whole-brain transparency following the optimized CLARITY protocol (Fig. 2). Considering that electrophoretic clearing may induce tissue damage, we used the passive clearing method at 60 °C to remove lipid bilayer in our study. We spent 14 days to obtain the whole transparent adult mouse brain. We found that the volume of the brain expanded after the passive clearing procedure, which was consistent with previous studies [25, 27]. After incubation in refractive index matching solution for 12 hours, the tissue returned to its original size and became completely transparent.

### Imaging of the vasculature in the whole mouse brain after CLARITY

**Figure 3.**
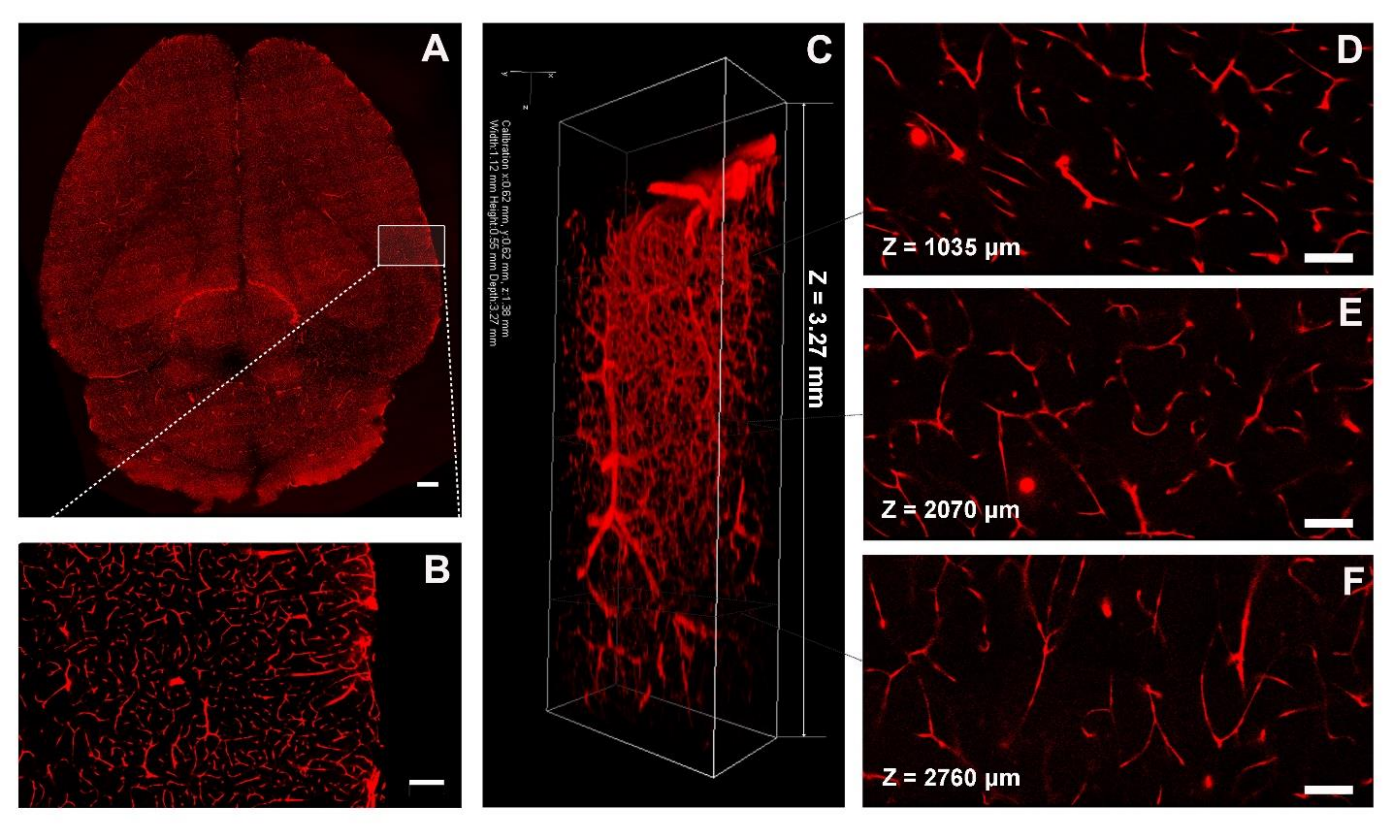
Imaging of vasculature in the whole mouse brain after CLARITY. A) Whole brain vessels of Tie2-RFP transgenic mouse were visualized using a confocal microscopy. Scale bar=1 mm. B) Magnification of white box region in (A). Scale bar=500 μm. C) 3D reconstruction of the vasculature in a whole, clarified mouse brain. Images were obtained using confocal microscopy equipped with a 40× water-immersion objective. The imaging volume was 1120 μm×550 μm×3270 μm with a voxel size of 0.62 μm×0.62 μm×1.38 μm. (D-F) Images at different brain depths (1035 μm, 2070 μm, and 2760 μm relative to the top imaging surface) using a 40× water-immersion objective with a voxel size of 0.62 μm × 0.62 μm × 1.38 μm. Scale bar=100 μm.

In CLARITY, hydrogel crosslinks with protein in the brain to preserve structural and molecular information for further imaging and analysis. Our results showed that brain vessels of Tie2-RFP transgenic mice were visualized at a high resolution using confocal microscopy (Fig. 3A). Red fluorescence was not quenched after CLARITY procedures. Furthermore, a imaging depth into the whole brain was 3270 μm, which was almost up to the working distance of a 40× water-immersion objective (Fig. 3C). At the same time, high signal-to-noise ratio images were obtained at different depths of the brain (1035 μm, 2070 μm, 2760 μm relative to the top imaging surface) using the 40× water-immersion objective with a voxel size of 0.62 μm×0.62 μm×1.38 μm (Fig. 3D, E and F).

### Imaging of vasculature in mouse brain slices with immunostaining after CLARITY

**Figure 4.**
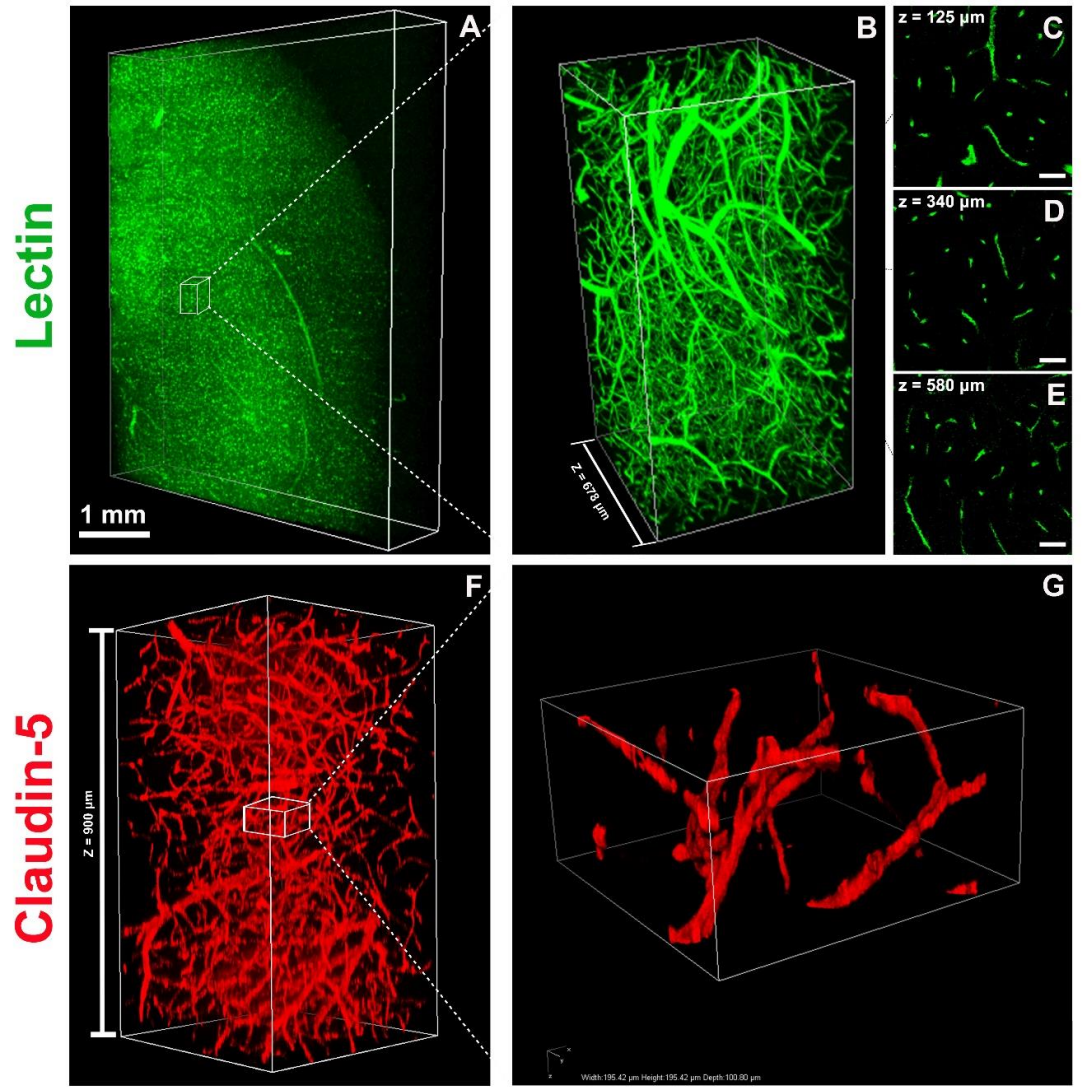
Imaging of vasculature in a 1-mm-thick mouse brain slice stained with lectin and claudin-5. A) 3D rendering of a 1-mm-thick wild-type C57BL/6J mouse brain slice stained with lectin-FITC. Images were obtained from confocal microscopy equipped with a 16× objective. The imaging volume was 4300 μm×5260 μm×880 μm, with a voxel size of 0.99 μm×0.99 μm×1.00 μm. Scale bar=1 mm. B) Magnification of the white box region in (A). The imaging volume was 1367 μm×682 μm×678 μm. C-E) Images at different depths of the brain slice from (B) are shown (125 μm, 340 μm, and 580 μm relative to the top imaging surface) using a 16× water-immersion objective with a voxel size of 0.99 μm×0.99 μm×1.00 μm. Scale bars=50 μm. (F) 3D rendering of a 1-mm-thick C57BL/6J mouse brain slice stained with claudin-5 antibody. Images were obtained using confocal microscopy equipped with a 16× objective. The imaging volume was 504 μm×504 μm×900 μm, with a voxel size of 0.99 μm×0.99 μm×1.00 μm. G) Magnification of white box region in (F). The imaging volume was 195 μm×195 μm×100 μm, with a voxel size of 0.99 μm×0.99 μm×1.00 μm.

CLARITY allows fluorochrome and antibody penetration into the deep brain by removing lipid bilayers. Lectin staining was performed to label vessel endothelium. Uniformly distributed labeling throughout brain slice is shown in Figure 4A and 4B. High signal-to-noise ratio images were also obtained at different depths of the brain slices (Fig. 4C-E). Claudin-5 is a tight junction protein that is expressed on endothelial cell of brain vessels [35]. Claudin-5 antibody was used to label brain vessels and penetrated into the whole thickness of the clarified C57BL/6J mouse brain slices (1 mm) (Fig. 4F and 4G).

### Visualization and quantification of vasculature in the whole mouse brain and brain slices after CLARITY

**Figure 5.**
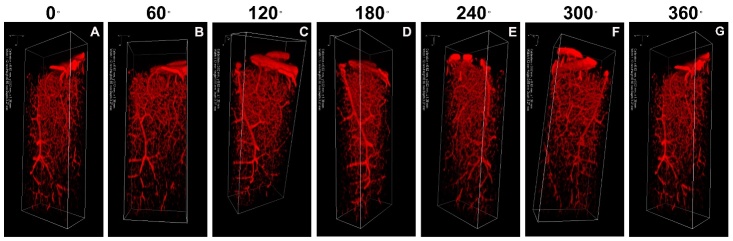
Visualization of vasculature in whole mouse brain after CLARITY. Visualization of 3D rendered images was performed using Vaa3D software and ewith 60°, 120°, 180°, 240°, 320°, and 360° anticlockwise rotations around the z-axis. Images were obtained using confocal microscopy equipped with a 40× water-immersion objective. The imaging volume was 1120 μm×550 μm×3270 μm, with a voxel size of 0.62 μm×0.62 μm×1.38 μm. A) 0°, B) 60° rotation, C) 120° rotation, D) 180° rotation, E) 240° rotation, F) 300° rotation, and G) 360° rotation.

**Figure 6.**
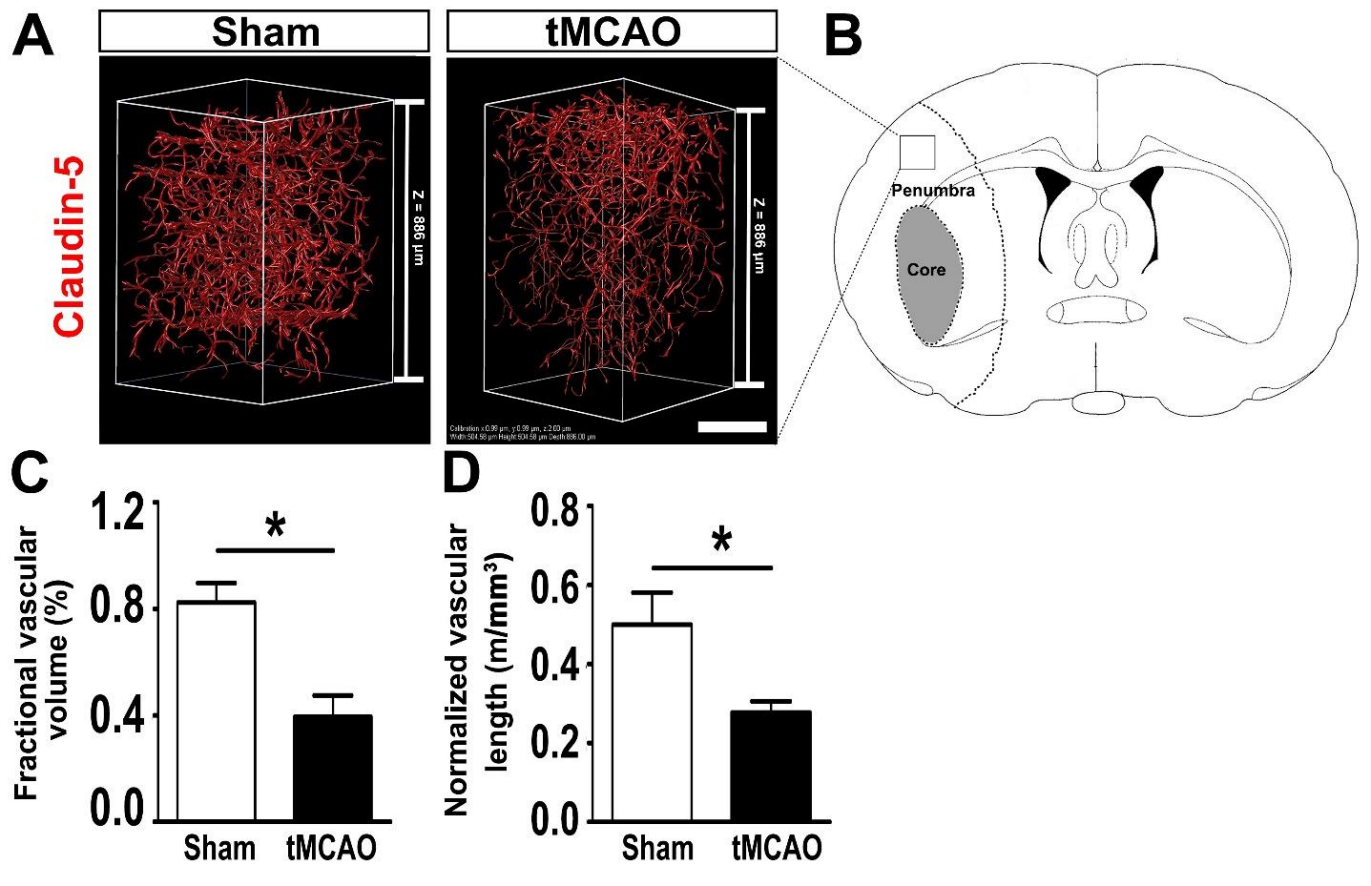
Visualization and quantification of the vasculature in the penumbra of tMCAO and sham mouse brain. A) Representative 3D image of the vasculature stained with claudin-5 in the penumbra of C57BL/6J mouse brain from the control and tMCAO group after 24 hours of reperfusion. The imaging volume was 504 μm×504 μm×886 μm, with a voxel size of 0.99 μm×0.99 μm×2.00 μm. Scale bar=250 μm. B) Mouse brain coronal section indicating core and penumbra region of the infarct area and the box indicating the area that was sampled. Quantification of the fractional vascular volume and the normalized vessel length in the controls and in the tMCAO group after 24 hours of reperfusion (C and D). Data are mean ± standard error, n=3 per group. *p < 0.05, tMCAO vs. control. tMCAO: transient middle cerebral artery occlusion.

By using image processing, visualization of 3D rendering images was performed, and 60°, 120°, 180°, 240°, 320°, and 360° anticlockwise rotations around the z-axis were shown in Figure 5. The quantitative results showed that the fractional vascular volume was 0.018 ± 0.004 mm^3^ per mm^3^. The normalized vascular length was 0.44 ± 0.04 m per mm^3^. The average diameter of the microvessels was 4.25 ± 0.08 μm. To further validate the CLARITY method for quantifying the vasculature in disease models, 1-mm-thick C57BL/6J mouse brain slices from the tMCAO group and the control group was cleared using the CLARITY technique and stained with claudin-5 for imaging. The fractional vascular volume and normalized length of the vessels were quantified in the ischemic penumbra 24 hours after tMCAO. We found that fractional vascular volume and average length of the vessels decreased significantly in the ischemic penumbra in the tMCAO mice compared with those in the sham group (*p* < 0.05, Fig. 6C and 6D).

## DISCUSSION

In our study, we mapped the vasculature in a whole mouse brain at cellular resolution using the CLARTY technique. Furthermore, we established optimized algorithms for vascular extraction and quantification that were compatible with CLARITY imaging. This imaging and analysis system provides a powerful tool for further evaluating structural the alterations of whole-brain vasculature in rodent disease models.

To obtain system-wide detailed information in the whole-brain, several clearing approaches have been developed [16, 18, 19, 24]. Each of these approaches has distinctive characteristics. Ertürk et al. [7] used the 3DISCO method to clear unsectioned spinal cord of an adult mouse. However, immunostaining was limited in this method [30]. Another study reported that Sca*l*eA2 made a adult mouse brain transparent without quenching the fluorescent dyes, but the clearing speed was slow (approximately 3 weeks), and the procedures led to potential protein loss [15]. The SeeDB technique cleared a fixed brain in a few days without tissue deformation and fluorescence quenching, but tissue browning was observed during clearing [19]. CUBIC provided another clearing method for whole brain imaging, even there was though potential protein loss accampanied with clearing [23]. As a newly developed clearing technique, CLARITY makes the tissue transparent in a relatively short time and preserved native molecules and structure [27]. Moreover, this method allows antibody to deeply penetrate into the brain by removing lipid bilayers, and permits rounds of staining. These advantages make this technique a promising method for structural and molecular interrogation of whole brain imaging. In our study, the imaging depth into the brain was 3270 μm using the confocal microscope, which was almost up to the working distance (3500 μm) of the 40× water-immersion objective. At the same time, high signal-to-noise ratio images were obtained at different brain depths. In addition, exogenous fluorescent molecules could penetrate into the whole thickness of the clarified slices (1mm) after 3-days of incubation at 37 °C. Our results showed that mapping the whole brain vasculature at a cellular resolution was achieved for not only an endogenous fluorescent reporter but also an exogenous fluorescent antibody using the CLARITY technique. This suggests that CLARITY is a useful approach for visualizing the spatial structure and distribution of vasculature in the whole mouse brain.

Furthermore, we optimized the image processing and analysis for CLARITY imaging over a large brain volume. In our study, we found that the brightness of the images decreased with the imaging depth. This phenomenon was also found in other clearing techniques and is associated with spherical aberration due to refractive index mismatch between the clearing reagent and the immersion medium [36].

Therefore, brightness homogenization was performed using histogram matching and illumination correction algorithms. Image preprocessing for brightness homogenization was also used in the MOST technique, which was a combination of block-face microscopy with tissue sectioning [12, 13]. Effective image identification and segmentation were also challenges for CLARITY image processing. Automatic filament algorithms developed from studies of kidney glomeruli were applied to detect and segment CLARITY ovarian vessels [37, 38]. However, algorithms to effectively identify cerebral vasculature were still undetermined. In our study, a 3D canny edge detection algorithm was selected, and this algorithm has been used in vessel segmentation of human brain 3D computed tomography (CT) images with high accuracy [39, 40]. We found that the processing procedures including 3D Canny edge detection also achieved high recall and precision value for CLARITY images.

In the present study, the fractional vascular volume and the normalized vascular length were selected as indicators of vascular density. Both vascular injury and angiogenesis induce changes in vascular density [41, 42]. Vessel diameter is another parameter that is used to assess vascular alteration. Changes in vascular diameter are closely related to the pathophysiologic mechanisms of many diseases [43, 44]. Magnetic resonance angiography has revealed many additional dilated vessels that were highly perfused in the penumbra area 48 hours after stroke [45]. Post-ischemic hyper-perfusion is thought to arise as a result of disturbed cerebral autoregulation. In previous studies, the value of the fractional vasculature volume ranged from 1% to 4% per mm^3^ [12, 34, 46-49]. The variation of the fractional vasculature volume in different studies might be attributed to tortuous and randomly organized capillary bed. Comparable to the values in previous studies, we found that the total vasculature volume accounted for approximately 1.8 % per mm^3^ of the imaging volume. Moreover, the average diameter of the microvessels was 4.1-4.4 μm in the current study. This was consistent with the diameter measured in thin brain sections, in cleared thick brain sections using a 60% sucrose solution as clearing reagent, and in an *in vivo* study [34, 46]. In addition, we quantified the 3-dimensional vascular tight junction pathology after tMCAO by using the CLARITY method. Consistent with the data acquired in 2 dimensions [35, 52], our 3-dimensional quantitative data also indicated decreased claudin-5 positive signals in the penumbra 24 hours after ischemic stroke. These results suggest that the structural alteration of vasculature in 3 dimensions could be evaluated by using the CLARITY method. At the same time, some limitations should be noted. During the CLARITY process, we noted that the tissue expanded after removing the lipid bilayers but returned to the original size after incubation in RIMS. This phenomenon was also shown in previous reports [26, 27]. Changes in tissue volume during the CLARITY procedures were estimated to be isotropic and reversible [25]. In addition, changes in the tissue size were encountered in other clearing methods and in the MOST technique [13, 15, 34]. This potential influence on the accuracy of quantitative analysis should be considered in the future.

In conclusion, we showed the utility of CLARITY in visualizing the vasculature of a whole adult mouse brain at cellular resolution. CLARITY-optimized image processing and analysis enables the further evaluation of the structural alteration in the vasculature after brain injury.
